# Sleep quality metrics combined with virtual reality motion parameters enhance early detection of mild cognitive impairment

**DOI:** 10.3389/fpsyt.2026.1727576

**Published:** 2026-04-22

**Authors:** Ruirui Zhang, Huaiqing Sun, Yaxuan Di, Hui Cao, Chengliang Zhang, Hongjun Yao, Hao Yan, Ding Ding, Qing He, Ting Wu

**Affiliations:** 1 Department of Neurology, The First Affiliated Hospital with Nanjing Medical University, Nanjing Medical University, Nanjing, China; 2 School of Computer Science and Engineering, Southeast University, Nanjing, China; 3 Department of Neurology, Xuzhou First People’s Hospital, The Affiliated Xuzhou Municipal Hospital of Xuzhou Medical University, Xuzhou, China

**Keywords:** digital markers, hand movement, mild cognitive impairment, sleep disorders, virtual reality

## Abstract

**Objective:**

Alzheimer’s disease (AD) is a progressive neurodegenerative disorder marked by cognitive and motor deficits. With its global prevalence increasing rapidly and no effective treatment available, early identification of high-risk individuals is critical. This study investigated the relationship between motor parameters extracted from virtual reality (VR) tasks, combined with sleep-related measures, and cognitive impairment in patients with mild cognitive impairment (MCI). Our goal was to determine whether integrating VR-derived digital markers with sleep quality metrics could provide an objective and clinically applicable tool for early detection.

**Methods:**

66 participants were recruited, including 28 healthy controls (HC) and 38 patients with MCI. Cognitive status was assessed using the Montreal Cognitive Assessment (MoCA) and Mini-Mental State Examination (MMSE). All participants performed two scenario-based VR tasks, during which task completion time, accuracy, and overall performance scores were recorded. Group differences were evaluated using independent-samples t-tests, and these behavioral features and sleep quality metrics were further incorporated into ROC analyze to assess predictive performance for distinguishing MCI from HC.

**Results:**

Compared with HC, patients with MCI reported significantly poorer sleep quality based on the Pittsburgh Sleep Quality Index (PSQI) and subdomains such as sleep latency and habitual sleep efficiency. In the VR tasks, MCI patients required more time and achieved lower accuracy than HC, consistent with MoCA and MMSE scores. Correlation analysis confirmed strong associations between VR performance metrics and cognitive test scores. Importantly, integrating VR-derived digital markers with sleep parameters yielded superior predictive accuracy for MCI (AUC = 0.863; sensitivity = 86.84%; specificity = 71.43%; p < 0.001) compared with single-modality models.

**Conclusion:**

VR-based cognitive and sensorimotor tasks, when combined with sleep quality assessments, offer a robust and noninvasive approach for the early identification of prodromal AD. This multimodal strategy holds promise for enhancing clinical decision-making and enabling timely interventions.

## 1 Introduction

Sleep is a fundamental biological process essential for brain health, supporting cellular repair and the clearance of metabolic waste (1). Sleep disorders, highly prevalent worldwide, disrupt circadian and homeostatic regulation and have been identified as major risk factors for cognitive decline (2–4). Disturbances such as insomnia, abnormal sleep duration, and sleep apnea reduce rapid eye movement (REM) sleep, impair glymphatic clearance, and exacerbate neuroinflammation in Alzheimer’s disease (AD) (5, 6). Importantly, sleep abnormalities emerge early, with patients with mild cognitive impairment (MCI) showing reduced slow-wave sleep and shortened total sleep duration (7). Therefore, early identification of sleep disorder-related cognitive impairment may delay or prevent dementia onset and facilitate more precise, individualized treatment.

Traditional screening tools, including the Montreal Cognitive Assessment (MoCA) and the Mini-Mental State Examination (MMSE), are widely used but may fail to capture subtle deficits in dynamic, real-world contexts (8–10). In addition to cognitive decline, patients with AD often exhibit fine motor impairments, such as slowed hand movements and reduced coordination, which are predictive of MCI-to-AD conversion (11–13). Digital markers—objective physiological and behavioral measures collected via digital devices—offer a promising complement to conventional assessments (14, 15). Among these, motor activity parameters are particularly valuable as they reflect early neural circuit vulnerability (16, 17).

Virtual reality (VR) technology offers unique advantages for clinical assessment, including high ecological validity, naturalistic user interaction, and real-time multimodal data capture (18, 19). Immersive VR environments provide patients with interactive, realistic experiences that enhance compliance and engagement while simultaneously generating objective behavioral metrics (20). These features have facilitated VR’s growing application in both the assessment and rehabilitation of cognitive disorders (21–23). Recent studies have demonstrated that VR-derived markers can improve the early detection of MCI and enhance cognitive and physical outcomes in AD (17, 24, 25). However, how best to integrate these multimodal digital markers with complementary factors, such as sleep quality, to create clinically actionable frameworks for early detection remains an open question.

To address this gap, we developed a novel VR-based cognitive assessment tool specifically designed to evaluate domains relevant to sleep impairment, such as sustained attention, episodic memory, and cognitive flexibility. By combining VR-derived digital markers with subjective sleep quality indices, we aimed to determine whether this multimodal approach could improve the early detection of MCI and provide a clinically applicable framework for screening cognitive decline.

## 2 Materials and methods

### 2.1 Study design and population

Participants were prospectively recruited at the First Affiliated Hospital of Nanjing Medical University between January and August 2025. A total of 70 participants were enrolled, of whom 4 were excluded due to incomplete VR data, resulting in a final analytical sample of 66 participants (94.3%). Missing data analysis using Little’s MCAR test confirmed that data were missing completely at random (χ² = 2.14, p = 0.34); therefore, complete-case analysis was employed. Outlier screening was performed using the boxplot criterion (1.5 × IQR) and Z-score threshold (|Z| > 3), and no extreme outliers were identified. The final sample comprised 28 HC and 38 patients with MCI. MCI diagnoses were confirmed independently by two neurologists based on established criteria (26). All participants underwent a standardized, multidisciplinary diagnostic assessment conducted by experienced neurologists (or neuropsychologists). The assessment included a comprehensive medical history review, a structured neurological examination, and a detailed neuropsychological battery covering multiple cognitive domains. MoCA was administered as a descriptive screening instrument only and was not used as a criterion for group assignment.

Inclusion required normal sensory perception and the ability to engage with the VR environment. Exclusion criteria were: (1) illiteracy or inability to read; (2) history of neurodegenerative or psychiatric disorders; and (3) prior dementia diagnosis or brain surgery. All participants provided written informed consent. This study complied with the Declaration of Helsinki (27) and was approved by the Institutional Review Board of the First Affiliated Hospital of Nanjing Medical University (2023-SR-916). Analyses were performed on anonymized datasets.

This study focused on individuals with MCI and excluded patients with AD, based on the following considerations: (1) MCI is a prodromal stage of AD and is considered a critical window for early intervention and screening. This study aimed to explore the value of VR-derived digital markers combined with sleep parameters for the early screening of MCI; (2) The VR tasks involved certain motor coordination and cognitive processing demands, which might be challenging for patients with moderate-to-severe AD to complete, potentially affecting data integrity and reliability; (3) Patients with AD often present with neuropsychiatric symptoms that could confound sleep assessments.

### 2.2 Neuropsychological and sleep assessments

Cognitive status was assessed using MoCA and MMSE, both administered in their validated Chinese versions to minimize language bias (28, 29). Sleep quality was assessed using three validated scales: the Pittsburgh Sleep Quality Index (PSQI) scale (30), the Athens Insomnia Scale (AIS) (31), and the Insomnia Severity Index (ISI) (32). All questionnaires were administered by trained assessors through one-on-one interviews to ensure that patients fully understood the meaning of each item. Particularly for individuals with cognitive impairment who may experience comprehension difficulties, we provided standardized explanations and, when necessary, sought confirmation from caregivers.

### 2.3 VR cognitive testing and assessment procedure

**Figure 1:**
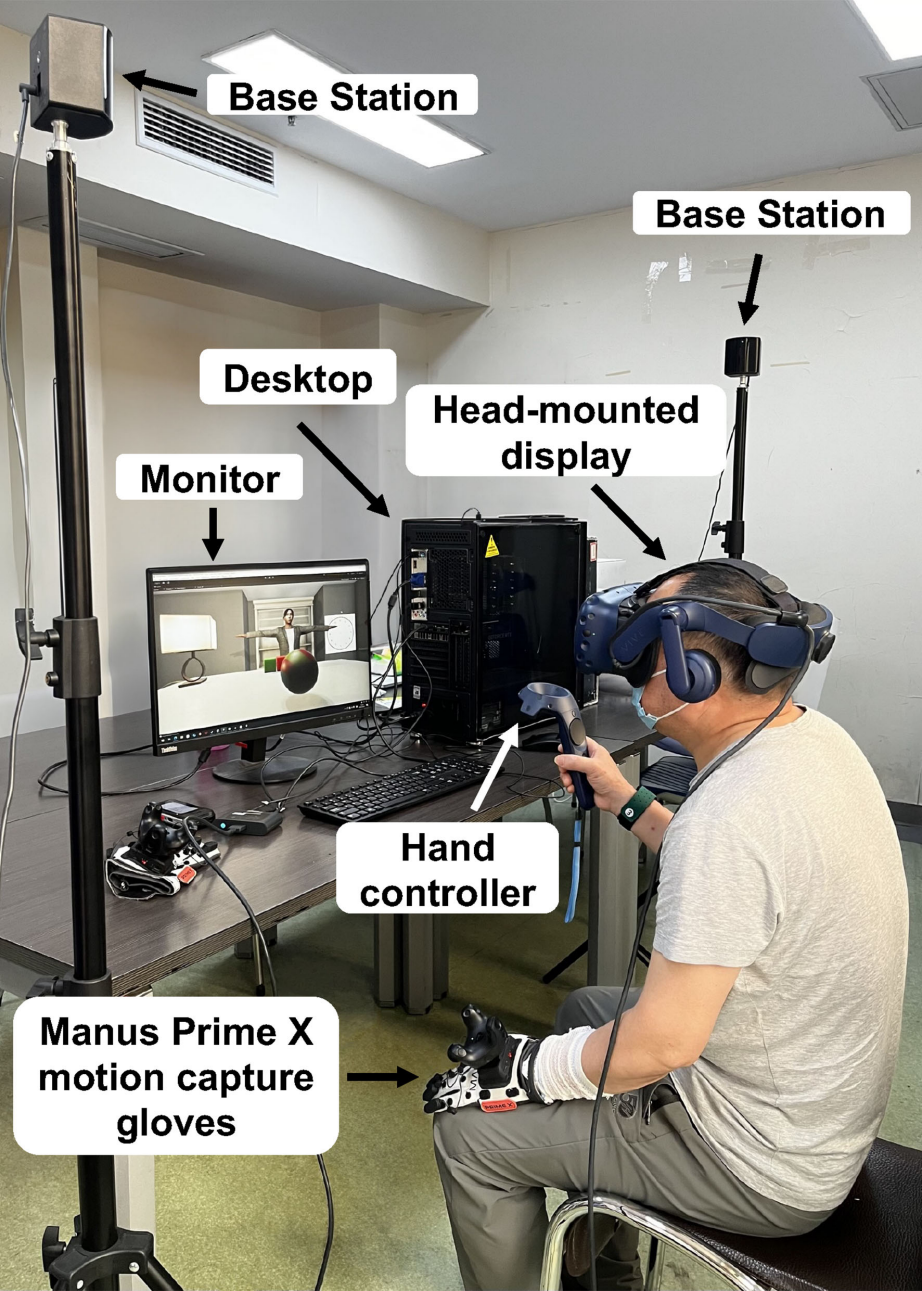
Experimental setup of the virtual cognitive task system. The VR classroom was equipped with a laptop, an HTC Vive Pro Eye head-mounted display with integrated eye tracking, Manus Prime X motion-capture gloves, and base stations. Participants remained seated during testing for safety.

VR-based assessments were conducted using a cognitive testing system developed by Professor Ding Ding’s team at Southeast University. Testing was conducted in a dedicated VR classroom (9.5 m × 7 m, ceiling height 4.5-5.5 m) equipped with a laptop (Intel i7 processor, 32 GB RAM, NVIDIA GeForce RTX 4070), an HTC Vive Pro Eye head-mounted display (90 Hz refresh rate; 1440 × 1600 resolution per eye) with integrated eye tracking, Manus Prime X motion-capture gloves, two base stations, and standard furniture (**Figure 1**). During testing, participants remained seated for safety. Eye movements were tracked by sensors embedded in the head-mounted display. A calibration procedure and a short practice session (5–10 minutes) were conducted before formal assessment to ensure comfort and familiarity. Each testing session lasted approximately 30 minutes. Two participants withdrew due to VR-induced dizziness and were excluded from analysis.

**Figure 2:**
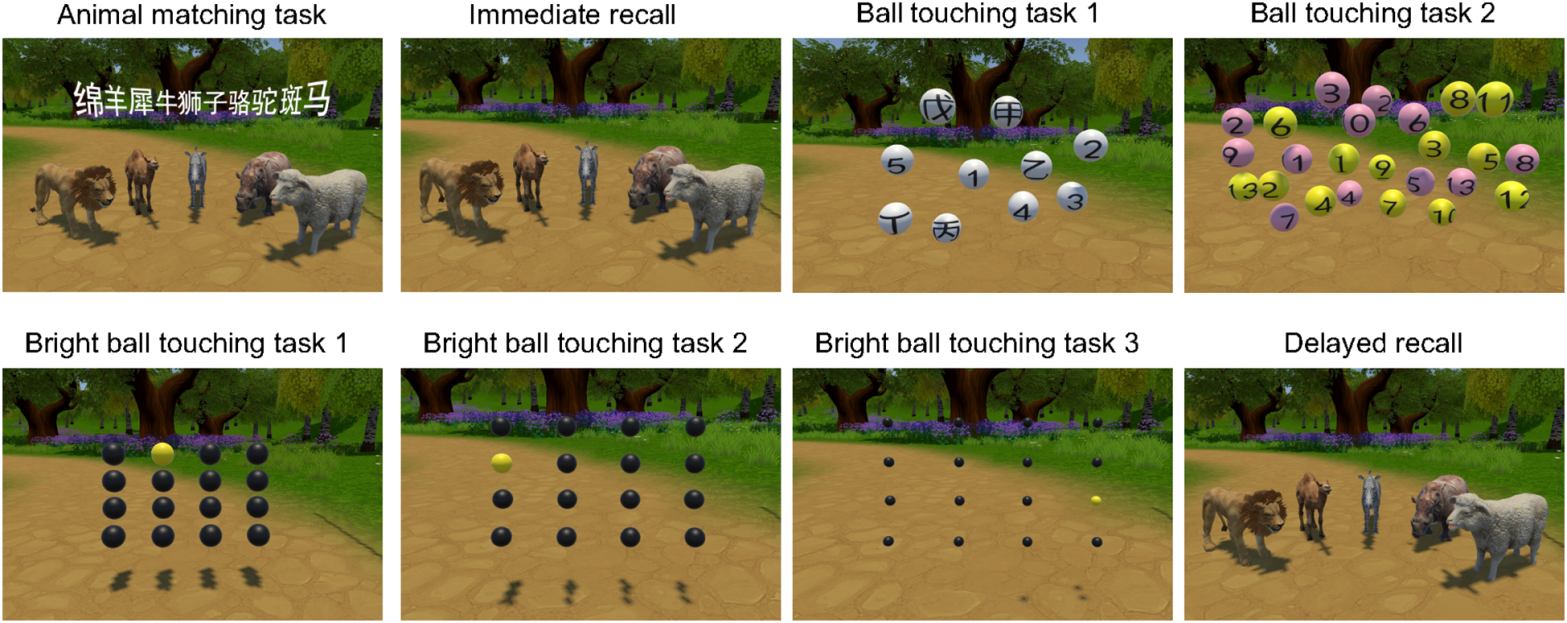
Sequential steps of scenario 1 in the VR cognitive function test. This scenario consisted of five tasks designed to assess naming, memory, visuospatial, and executive functions.

**Figure 3:**
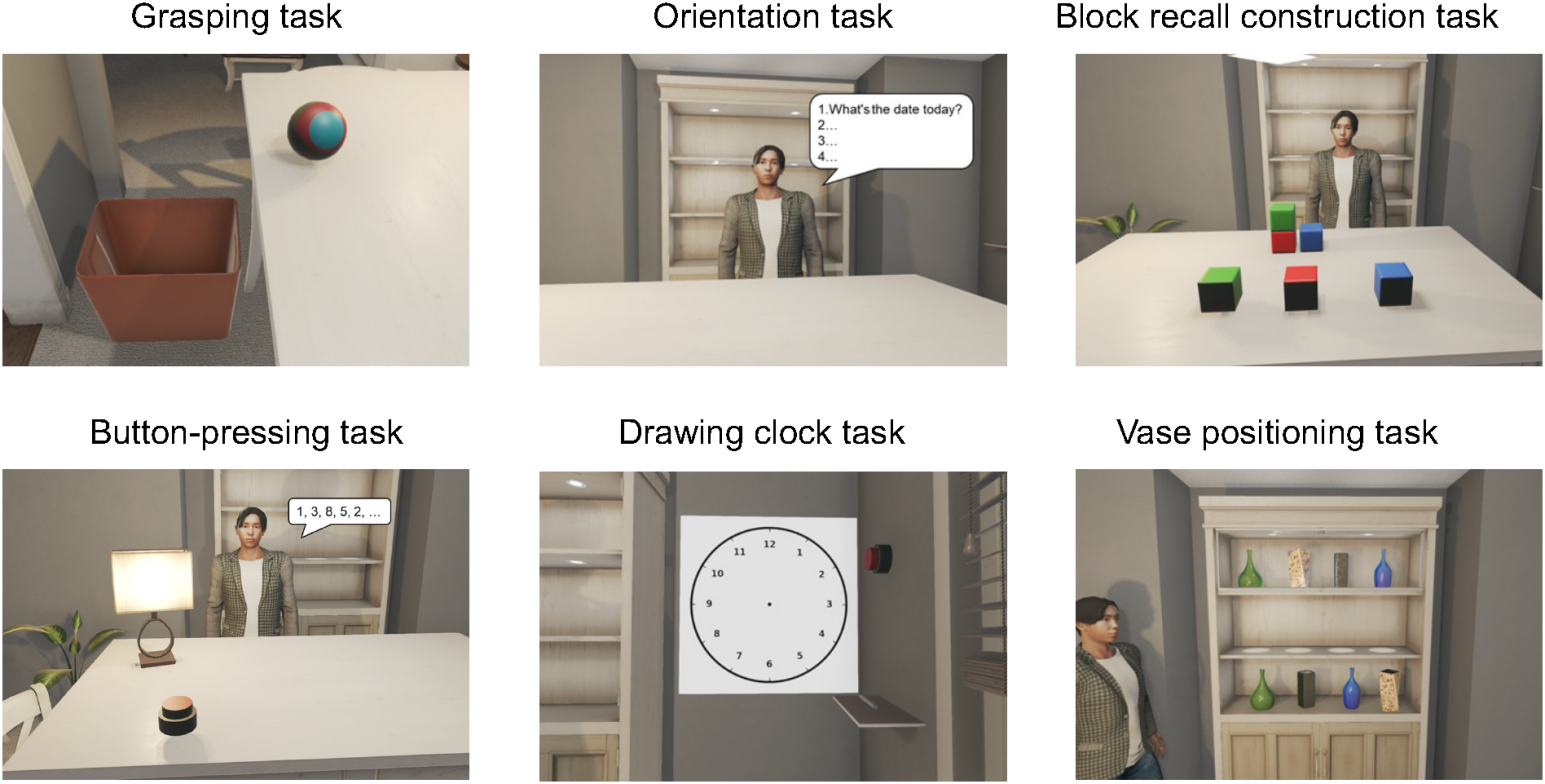
Sequential steps of scenario 2 in the VR cognitive function test. This scenario consisted of six tasks targeting memory, visuospatial, executive, orientation, and attention domains.

The system comprised two scenarios with progressively increasing difficulty based on cognitive load and motor complexity. Scenario 1 included five tasks assessing animal matching, immediate and delayed recall, and ball touching; Scenario 2 included six tasks evaluating grasping, orientation, block recall construction, button pressing, clock drawing, and vase positioning (**Figures 2**, **3**). To balance learning and fatigue effects while maintaining standardized testing, the following sequence control strategies were implemented: (1) randomization between scenarios—participants were randomly assigned to one of two scenario order conditions; (2) fixed order within scenarios—tasks were presented in a fixed order of increasing difficulty; and (3) familiarization phase—all participants completed a practice trial prior to formal testing.

### 2.4 Statistical analyses

Baseline demographic and clinical characteristics were compared between groups using independent-samples t-tests for continuous variables with normal distributions and chi-square tests for categorical variables. VR task performance and sleep scale scores were compared using independent two-tailed t-tests. Correlation heatmaps were generated to visualize relationships among digital markers, sleep indices, and cognitive test results.

A binary logistic regression model was constructed to evaluate the discriminative ability of combined VR and sleep parameters for mild cognitive impairment. Predictive performance was assessed using receiver operating characteristic curve (ROC) analysis. Subsequently, 5-fold cross-validation was performed to evaluate the stability and generalizability of the model.

All analyses were conducted using SPSS version 26.0 (IBM Corp, Armonk, NY, USA) and R version 4.4.2 (R Foundation for Statistical Computing, Vienna, Austria). Missing data mechanism was assessed using Little’s MCAR test. Outliers were identified using the boxplot method and Z-score method. Statistical significance was set at P < 0.05 (two-tailed).

## 3 Result

### 3.1 Baseline characteristics and neuropsychological test of participants

Our results showed no significant differences in sex, age, or educational level between the HC group and patients with MCI (**Table 1**). However, the total scores of the neuropsychological tests MoCA and MMSE were significantly lower in the MCI group compared with the HC group (25.25 ± 4.033 vs. 19.42 ± 4.763 and 26.86 ± 3.217 vs. 23.16 ± 3.929, both p < 0.001; **Table 1**). Moreover, the two groups differed significantly in several MoCA subdomains, including Visuospatial/Executive functions, Attention, Language, Abstraction, Delayed Memory, and Orientation, whereas in the MMSE, significant differences were only observed in the Orientation, Attention and Calculation, and Repetition subitems.

**Table 1:** Baseline demographic and neuropsychological characteristics of participants.

Characteristics	HC(n=28)	MCI(n=38)	P-value
Basic demographic characteristics			
Age (years), mean (SD)	62.43 (9.818)	62.18 (11.992)	0.93
< 65, n (%)	60.71	65.78	0.217
≥ 65, n (%)	39.29	34.22	0.683
Male, n (%)	35.71	47.37	0.451
Educational level (years), mean (SD)	12.21 (4.841)	10.29 (4.741)	0.111
Diabetes, n (%)	7.14	18.42	0.187
Hypertension, n (%)	25	31.58	0.56
Hyperlipidemia, n (%)	7.14	0	0.094
Cardiovascular disease, n (%)	3.57	5.26	0.744
Cerebrovascular disease, n (%)	3.57	15.79	0.111
Obstructive Sleep Apnea Syndrome, n (%)	7.14	2.63	0.385
Depression Disorder, n (%)	0	5.26	0.218
Neuropsychological tests			
MoCA total score, mean (SD)	25.25 (4.033)	19.42 (4.763)	<0.001
Visuospatial/Executive functions, mean (SD)	4 (1.155)	2.53 (1.289)	<0.001
Naming, mean (SD)	2.75 (0.585)	2.55 (0.602)	0.188
Attention, median (IQR)	6 (6-6)	6 (4-6)	0.001
Language, mean (SD)	2.21 (0.787)	1.61 (0.855)	0.004
Abstraction, mean (SD)	1.71 (0.535)	1.24 (0.786)	0.005
Remote memory, mean (SD)	2.82 (1.565)	1.16 (1.586)	<0.001
Orientation, median (IQR)	6 (5-6)	5 (4-6)	0.043
MMSE total score, mean (SD)	26.86 (3.217)	23.16 (3.929)	<0.001
Orientation, mean (SD)	9 (1.155)	7.03 (2.224)	<0.001
Recent memory, mean (SD)	3 (3-3)	3 (3-3)	0.391
Attention and calculation, mean (SD)	4.54 (0.922)	3.66 (1.494)	0.005
Remote memory, mean (SD)	1.93 (1.052)	1.5 (1.225)	0.429
Naming, median (IQR)	2 (2-2)	2 (2-2)	0.391
Repetition, median (IQR)	1 (1-1)	1 (1-1)	0.01
Reading, median (IQR)	1 (1-1)	1 (1-1)	0.177
3-Stage Command, median (IQR)	3 (3-3)	3 (2-3)	0.349
Writing, median (IQR)	1 (1-1)	1 (0.75-1)	0.18
Drawing, median (IQR)	1 (1-1)	1 (1-1)	0.867

Values are presented as mean ± standard deviation (SD) for continuous variables and number (percentage) for categorical variables. HC, Healthy Controls; MCI, Mild Cognitive Impairment; MoCA, Montreal Cognitive Assessment; MMSE, Mini-Mental State Examination.

### 3.2 Between-group comparisons of sleep quality and VR task performance

#### 3.2.1 Sleep quality parameters

As the scores of the sleep quality assessment scale were normally distributed, independent-sample two-tailed t-tests were used to evaluate differences between the two groups. As shown in **Table 2**, the total PSQI scores were significantly higher in the MCI group than in the HC group (8.61 ± 4.946 vs. 5.14 ± 4.344, p = 0.004), with significant differences observed in the components of sleep latency, sleep duration, and habitual sleep efficiency. Moreover, the AIS and ISI scores also showed significant differences between the two groups.

**Table 2:** Subjective sleep quality measures in MCI patients and HC.

Sleep parameters	HC(n=28)	MCI(n=38)	P-value
PSQI, mean (SD)	5.14 (4.344)	8.61 (4.946)	0.004
Subjective sleep quality, mean (SD)	0.96 (0.838)	1.37 (0.942)	0.076
Sleep latency, mean (SD)	0.79 (1.641)	1.76 (1.324)	0.009
Sleep duration, mean (SD)	0.75 (0.799)	1.26 (1.155)	0.037
Habitual sleep efficiency, mean (SD)	0.36 (0.78)	1.29 (1.183)	<0.001
Sleep Disturbances, mean (SD)	1.18 (0.612)	1.32 (0.662)	0.393
Use of Sleep Medication, mean (SD)	0.14 (0.591)	0.13 (0.578)	0.938
Daytime Dysfunction, mean (SD)	0.96 (1.036)	1.47 (1.156)	0.069
AIS, mean (SD)	3.36 (3.88)	7.03 (5.395)	0.002
ISI, mean (SD)	5.25 (5.535)	9.18 (7.526)	0.017

Data are expressed as mean ± SD. Higher scores indicate poorer sleep quality or more severe insomnia. HC, Healthy Controls; MCI, Mild Cognitive Impairment; PSQI, Pittsburgh Sleep Quality Index; AIS, Athens Insomnia Scale; ISI, Insomnia Severity Index.

#### 3.2.2 Virtual cognitive test performance

Independent-sample two-tailed t-tests were conducted to evaluate differences in virtual cognitive function test performance between the HC and MCI groups (**Table 3**). In Virtual Task 1, significant differences were observed between groups in the following measures: time to completion of the animal matching task; time to completion and accuracy of the immediate recall task; time to completion of the ball touching task; time to completion of the bright ball touching task; and time to completion and accuracy of the delayed recall task (all p < 0.05). In Virtual Task 2, patients with MCI showed significantly longer completion times than HC in the grasping task, the drawing clock task, and the vase positioning task (p < 0.05; **Table 3**).

**Table 3:** Performance on virtual cognitive tasks in MCI patients and HC.

Virtual task test feature	HC(n=28)	MCI(n=38)	p-value
Virtual task 1, mean (SD)			
Animal matching task			
Time to completion (seconds)	77.71 (43.3)	135.25 (108.66)	0.005
Accuracy(%)	0.79 (0.26)	0.72 (0.28)	0.321
Immediate recall			
Time to completion (seconds)	54.1 (31.13)	96.58 (73.04)	0.002
Accuracy(%)	0.65 (0.24)	0.5 (0.22)	0.011
Ball touching task			
Time to completion (seconds)	245.82 (124.35)	450.74 (313.7)	0.001
Numerical sequence accuracy(%)	0.72 (0.41)	0.6 (0.23)	0.151
Color sequence accuracy(%)	0.43 (0.45)	0.32 (0.27)	0.223
Bright ball touching task			
Time to completion (seconds)	96.75 (30.07)	141.51 (115.38)	0.027
Large ball-accuracy(%)	0.71 (0.37)	0.75 (0.17)	0.612
Middle ball-accuracy(%)	0.76 (0.37)	0.80 (0.14)	0.519
Small ball-accuracy(%)	0.79 (0.36)	0.79 (0.32)	0.993
Delayed recall			
Time to completion (seconds)	44.01 (18.5)	62.52 (30.68)	0.003
Accuracy(%)	0.63 (0.22)	0.48 (0.3)	0.037
Virtual task 2, mean (SD)			
Grasping task time (seconds)	31.83 (12.94)	45.13 (28.49)	0.014
Orientation task score	3.36 (1.06)	2.84 (1.13)	0.065
Block recall construction task			
Time to completion (seconds)	111.94 (47.38)	116.73 (53.53)	0.707
Score	1.86 (1.11)	1.5 (1.2)	0.223
Button-pressing task accuracy(%)	0.91 (0.13)	0.86 (0.15)	0.256
Drawing Clock Task			
Time to completion (seconds)	50.44 (19.53)	73.89 (46.23)	0.007
Score	1.25 (0.8)	0.82 (0.73)	0.025
Vase positioning task (seconds)	97.58 (41.37)	137.79 (81.67)	0.011

Task performance is reported as completion time (seconds) and accuracy (%), expressed as mean ± SD. Significant group differences were assessed using independent-samples t-tests. HC, Healthy Controls; MCI, Mild Cognitive Impairment.

### 3.3 Correlations between sleep and VR markers with neuropsychological test

**Figure 4:**
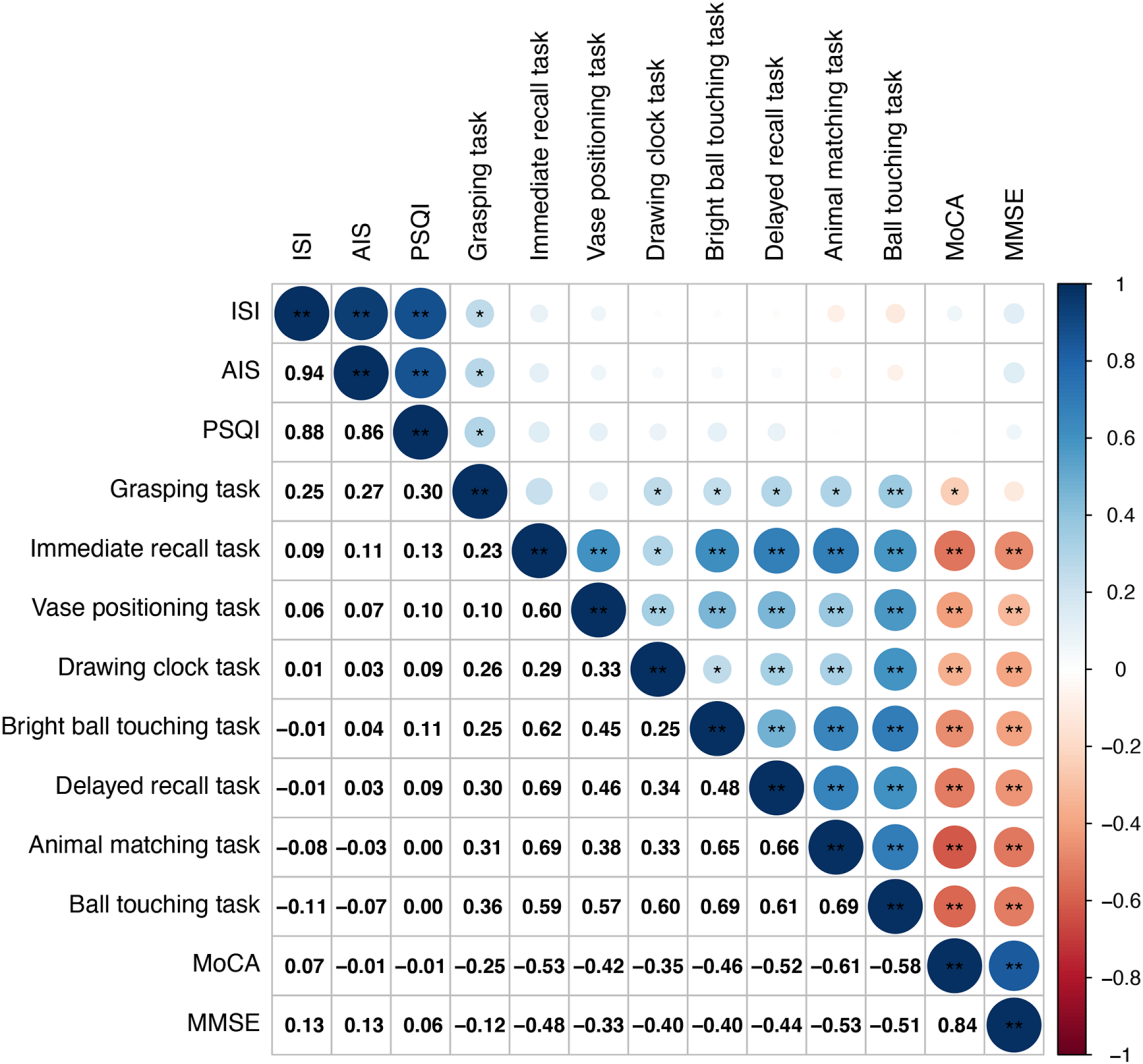
Correlation heatmap showing relationships among VR-derived digital biomarkers, sleep quality indices, and neuropsychological test results. HC: Healthy controls; MCI: Mild cognitive impairment.

We performed a Pearson correlation heatmap analysis on markers that showed statistical significance to explore relationships among VR task-derived digital markers (animal matching task, immediate recall task, ball touching task, bright ball touching task, delayed recall task, grasping task, drawing clock task, and vase positioning task), sleep-related markers, and neurocognitive function test results (**Figure 4**).

Our study revealed that digital markers derived from the virtual cognitive test, including the animal matching, immediate recall, ball touching, bright ball touching, delayed recall, drawing clock, and vase positioning tasks, were significantly negatively correlated with MoCA and MMSE scores, suggesting that longer completion times on these VR tasks were associated with poorer cognitive function (all |r| > 0.3, P < 0.05; **Figure 4**). Notably, the grasping task exhibited strong correlations with all sleep-related parameters, particularly the PSQI, whereas the associations between sleep-related parameters and MoCA or MMSE scores were relatively weak.

### 3.4 Performance and validation of the multimodal predictive model

We constructed a binary logistic regression model using the Enter method to evaluate the predictive value of combining sleep and VR-derived parameters for the early screening of MCI. Predictive performance was assessed using ROC analysis.

**Figure 5:**
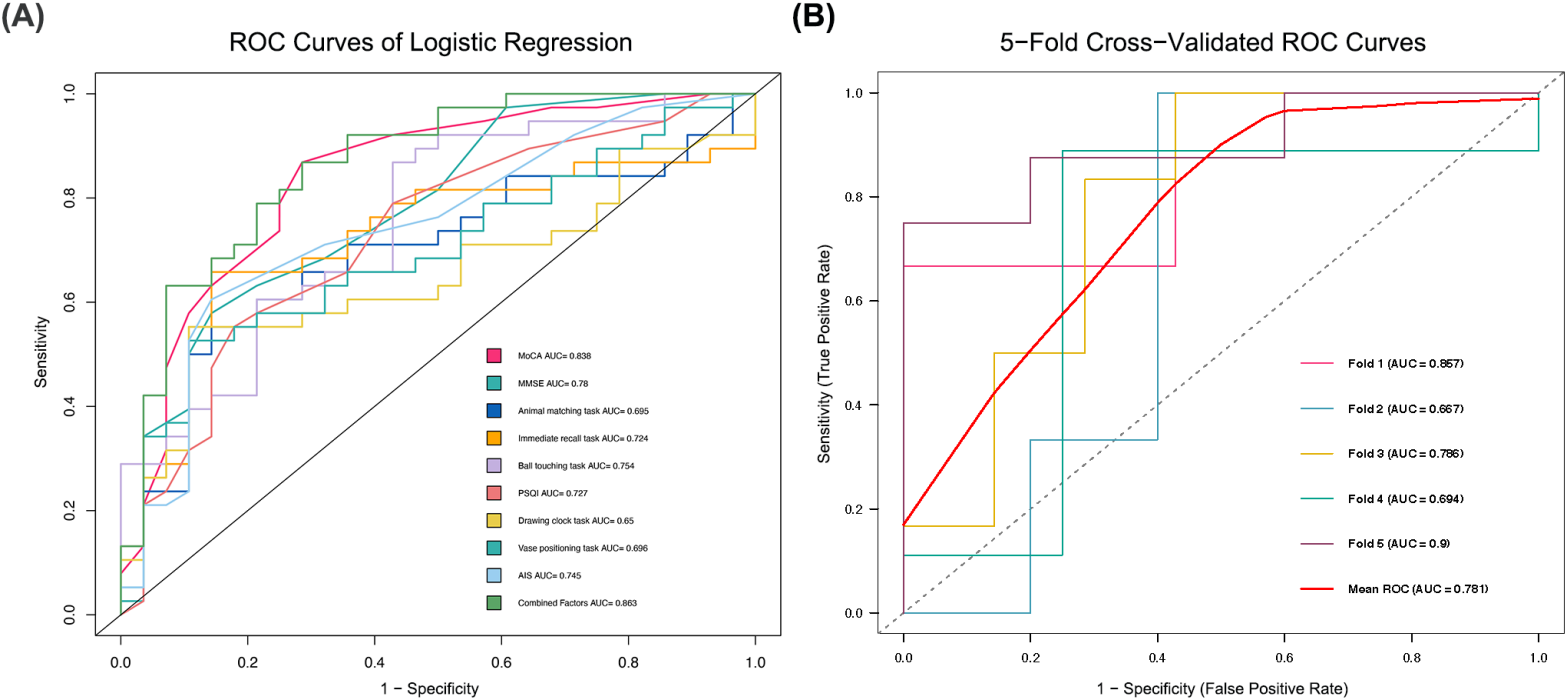
**(A)** ROC curves of logistic regression demonstrating the predictive performance of VR-derived digital biomarkers, sleep quality indices, and their multimodal integration for the detection of MCI. **(B)** ROC curves of the 5-fold cross-validation.

As shown in **Table 4** and **Figure 5 A**, the predictive model combining the ball touching task from the virtual cognitive test with the PSQI and AIS demonstrated the best diagnostic performance for patients with MCI (sensitivity 86.84%, specificity 71.43%, AUC = 0.863; P < 0.001). Notably, regardless of which single risk factor was selected, the predictive performance of individual parameters was inferior to that of the combined model.

**Table 4:** The predictive value of VR task and sleep parameters for MCI.

Variables	Best predicted value	Sensitivity (%)	Specificity (%)	AUC (95% CI)	*P*
MoCA	24.5	86.84	71.43	0.838 (0.736-0.939)	<0.001
MMSE	24.5	57.9	85.71	0.780 (0.666-0.893)	<0.001
PSQI	7.5	55.26	82.14	0.727 (0.602-0.852)	<0.001
AIS	2.5	68.8	53.3	0.638 (0.476-0.799)	<0.001
Animal matching task	94.46	55.26	85.71	0.695 (0.566-0.825)	0.003
Immediate recall task	61.49	65.79	85.71	0.724 (0.596-0.852)	0.001
Ball touching task	234.17	86.84	57.14	0.754 (0.635-0.872)	<0.001
Drawing clock task	56.58	55.26	89.28	0.650 (0.515-0.785)	0.028
Vase positioning task	119.76	52.63	89.28	0.696 (0.569-0.824)	0.002
Combined	Ref.	86.84	71.43	0.863 (0.771-0.954)	<0.001

VR, Virtual Reality; MCI, Mild Cognitive Impairment; MoCA, Montreal Cognitive Assessment; MMSE, Mini-Mental State Examination; PSQI, Pittsburgh Sleep Quality Index; AIS, Athens Insomnia Scale. AUC, area under the curve.

To assess model stability and generalizability, 5-fold cross-validation was performed. The ROC curves of the 5-fold cross-validation are presented in **Figure 5B**. The AUC values for the individual validation folds were 0.857, 0.667, 0.810, 0.694, and 0.913, respectively. The aggregated mean ROC curve across folds, depicted as the bold red line in **Figure 5B**, yielded an AUC of 0.781. The cross-validated mean AUC was 0.781 (SD = 0.101), with a 95% CI ranging from 0.656 to 0.906. These results indicate that the multimodal logistic regression model exhibits moderate-to-high discriminative performance in predicting MCI. The relatively low inter-fold variation in AUC values suggests robust model stability and a reduced likelihood of overfitting under the current data distribution. Thus, the combined approach of VR-derived digital markers and sleep parameters provides the most promising predictive outcomes, which are slightly superior to the diagnostic criteria for MCI represented by MoCA and MMSE. Additionally, VR-derived digital markers alone showed good predictive performance, while sleep parameters alone performed relatively lower.

## 4 Discussion

The primary objective of this study was to determine whether integrating VR-derived digital markers with sleep parameters could improve early detection of MCI. Our results confirmed that MCI patients reported poorer subjective sleep quality, with higher PSQI, AIS, and ISI scores, and performed significantly worse on VR-based tasks, showing longer completion times, higher error rates, and lower accuracy. Together, these findings demonstrate that both sleep quality indices and VR-derived behavioral metrics reliably distinguished MCI from healthy controls. Moreover, their combination achieved superior diagnostic performance compared with either modality alone or conventional screening tools.

A key finding is that the multimodal predictive model achieved high diagnostic accuracy (AUC = 0.863; sensitivity = 86.84%; specificity = 71.43%). Its performance surpasses that of models based solely on sleep parameters and VR-derived digital markers. For example, while the ball-touching task alone yielded high sensitivity but lower specificity (57.14%), and PSQI alone showed strong specificity (82.14%) but lower sensitivity (55.26%), their integration balanced these strengths and limitations, producing a more robust diagnostic tool. This demonstrates the complementarity of VR and sleep-related markers.

Our study also highlights the unique contributions of VR-based tasks in uncovering cognitive processes not fully captured by traditional tests. The ball touching task of VR digital markers in this study exhibited high sensitivity with 86.84%, indicating their ability to accurately classify healthy controls, a finding that is consistent with the results of several previous studies (17, 23, 33). The animal-matching and ball-touching tasks assessed working memory, visuospatial ability, attention, and information processing speed, while the delayed recall and executive memory paradigms were strongly correlated with neuropsychological performance. Moreover, working memory, which involves temporarily retaining information for manipulation and decision-making, is a key cognitive process in virtual cognitive function assessment and has been shown to be significantly impaired in patients with AD (34). Interestingly, the grasping task correlated more strongly with sleep indices than with MoCA or MMSE, suggesting that VR tasks may reveal subtle links between sleep disturbances and motor-cognitive integration. This means that participants with sleep disturbances tend to show poorer attention and executive function, longer completion times, lower accuracy rates, and fewer points in the virtual cognitive tests. Considering the key role of sleep factors in memory formation and learning, this observation is consistent with previous research findings (35).

Sleep abnormalities are widely recognized as both early manifestations and risk factors for cognitive decline, contributing to impaired memory consolidation, glymphatic dysfunction, and neuroinflammation (36). PSQI in our study showed excellent specificity for MCI prediction. However, VR markers alone demonstrated high sensitivity but limited specificity, while sleep markers showed the reverse. Their integration therefore provides a more balanced and clinically valuable approach for early detection. Beyond diagnostic accuracy, the integration of VR and sleep markers offers advantages over traditional imaging or fluid biomarkers, which typically achieve pooled sensitivities and specificities of 70% and 80% (37), respectively, but are costly and invasive. By contrast, VR and sleep based assessments are cost-effective, noninvasive, and scalable, making them well suited for community-level screening. For example, high-risk individuals identified using this multimodal framework in community centers could then be referred for confirmatory diagnostic procedures in hospitals, enabling timely intervention while reducing healthcare burden.

Our findings demonstrate that combining VR−based motor−cognitive parameters with sleep measures significantly improves MCI prediction accuracy, suggesting shared neurobiological substrates. Sleep disturbances impair prefrontal and hippocampal function (38–41)—core regions supporting executive function, spatial navigation, and memory—which are precisely the cognitive processes engaged by our VR tasks. Additionally, sleep fragmentation affects attention, processing speed, and motor coordination (42), and impairs memory consolidation essential for procedural and spatial learning in VR (43). Thus, sleep quality likely modulates VR task performance via prefrontal-hippocampal circuit integrity. Future studies integrating neuroimaging and electrophysiology are needed to clarify these neural pathways.

### 4.1 Strengths and limitations

The strengths of this study include the application of a novel VR-based cognitive testing platform, the integration of sleep and cognitive measures, and the demonstration of multimodal predictive modeling. Nonetheless, some limitations should be noted. First, this single-center study had a relatively small sample size; future multicenter studies with larger samples are needed to confirm generalizability. Second, sleep quality was assessed using subjective questionnaires, which may introduce recall bias. Future studies should incorporate objective measures such as polysomnography (PSG) or portable sleep monitoring devices to objectively quantify sleep parameters. Third, although VR-derived measures correlated with neuropsychological performance, further research is warranted to investigate their underlying neurobiological basis using imaging and fluid biomarkers.

## 5 Conclusion

In summary, this study provides evidence that combining VR-derived digital markers with sleep quality indices offers a powerful approach for early detection of MCI. The multimodal predictive model demonstrated superior performance compared with traditional tools, highlighting its potential as a rapid, noninvasive, and scalable screening strategy. By capturing both subtle motor-cognitive impairments and sleep-related risk factors, this framework may support individualized clinical decision-making, improve early intervention, and ultimately help reduce the societal burden of dementia.

## Data Availability

The raw data supporting the conclusions of this article will be made available by the authors, without undue reservation.
